# Book Review: The Interoceptive Mind: From Homeostasis to Awareness

**DOI:** 10.3389/fpsyg.2020.637640

**Published:** 2021-01-28

**Authors:** Jing Zhang, Da Dong

**Affiliations:** ^1^School of Marxism, Institute of Philosophy, Hangzhou Dianzi University, Hangzhou, China; ^2^School of Medicine, Technische Universität Dresden, Dresden, Germany; ^3^Department of Philosophy, Center for the Study of Language and Cognition, Zhejiang University, Hangzhou, China

**Keywords:** interoception, homeostasis, awareness, embodied cognitive science, sense of self

To understand the nature of mind, cognitive scientists of all theoretical strands may benefit from considering experimental evidence of cognition (for example, perception, attention, memory) alongside phenomenology of bodily self (or conscious) feelings (proprioceptive, exteroceptive, and interoceptive). Roughly, the former dimension of scientific studies of mind seems to have found its legitimacy; yet, at first glance, the latter could still not be exonerated completely. That is to say, as far as the scientifically evidence-based account of endogenously generated interoceptive feeling is concerned, we will have come a long way toward buttressing its legitimacy. *The Interoceptive Mind: From Homeostasis to Awareness* can be seen as a guide to scientific studies of interoception in all of the cases so far. In particular, the essays collected in this book shed light on multisensory integration of interoceptive and exteroceptive feelings (and even self-conscious feelings) across widespread disciplines.

As suggested in its title, the book addresses the difficulties of integrating various dimensions relevant to feelings—from the biological notion of self to the interoceptive origins of self-awareness. The book divides into four sections, and each chapter is worthy of careful attention.

Part I introduces the historical background of research in interoception. Based on a case report, the authors elaborate on why the autonomic nervous system is the dominant efferent system and a fine-constructed afferent system. They point out that interoception and then advanced brain functions are dependent upon each other. Thus, a full-fledged theory of self might result from interactions (or rather, “tradeoffs”) of bottom-up sensory input and top-down predictions. This point is key to the authors' argument.

Part II focuses on the newest research in cognitive neuroscience and experimental psychology and emphasizes interoception's importance to embodied cognitive science by resorting to a popular predictive processing framework. These chapters are the following: Chapter 2 affirms the significance of the body for self-awareness from the perspective of interoceptive predictive processing (IPP) and proposes that interoception serves the stability of self-consciousness. Chapter 3 attempts to assume that the integration of visceral signals probably produces a subject-centered frame of reference, which could be seen as the basis of bodily self. Chapter 4 explores how interoception influences temporal perception. Chapter 5 presents the current debates of “gut feelings” and the brain–gut axis hypothesis. Chapter 6 provides reliable evidence for a more general definition of interoception by scrutinizing the effect of painful/pleasant touch on the surface of the skin relating to bodily-self representation. It is a pity that researches on the influence of interoception on theory of mind, decision-making, and emotion regulation are not mentioned here (Gao et al., 2019).

Part III concerns interoception with physical and mental health. Chapter 7 reveals a close relevance between interoception and emotion via the IPP framework. Chapter 8 draws a distinction between two “worlds” in our brains: the “inner world” of the body and the “outer world” where the body lives (p. 145). Chapter 9 uses examples of anorexia nervosa and bulimia nervosa to illustrate that the dysfunction of interoception is the root cause of maladaptive eating behaviors. Chapter 10 focuses on the dysfunctions of interoceptive mechanisms in various neurological disorders, such as Alzheimer's disease and Parkinson's disease. The authors propose a future direction on understanding manifestations of cerebral damages through the neurology of interoception. Chapter 11 suggests that medically unexplained symptoms could have perceptible symptoms as their root cause. A more effective methodology of tackling symptoms (those are not related to physiological dysfunctions) might be indirect interoceptive differentiation training (p. 223). Chapter 12 confirms that interoception is the core component of subjective happiness valuation and then designs a respiration-based task for characterizing interoception concerning mental health. It would be more interesting if this section involves more discussions about possible ways of training and improving interoception to promote mental health.

Lastly, Part IV promotes and integrates multidisciplinary research in philosophy. Chapter 13 points out that it is necessary to distinguish between physiological arousal and experiential arousal. They also address that physiological arousal cannot be reduced to a uniform pattern of sympathetic activation; experiential arousal cannot be reduced to visceral sensations of the autonomic nervous system as well. Chapter 14 reconsiders how interoception contributes to consciousness and self. Chapter 15 argues that homeostasis and allostasis are “complementary strategies for sustaining biological viability” (p. 289) and constructing interoceptive inference. Chapter 16 sums up several essential characteristics of subjectivity and then indicates the consistency between these characteristics and the non-topological dimension of interoception. The last chapter provides a phenomenological investigation of internal bodily experience and presents how the external world influences the bodily experience. This part reflects the editors' acknowledgment of the standpoint that a comprehensive understanding of interoception requires us to integrate the first-person perspective with the third-person perspective within the realm of consciousness science (Ceunen et al., 2016).

From our perspective, the book's major shortcoming is as follows: the concept of “interoception” lacks a more precise and uniform treatment. Nevertheless, the book might beg the reader's pardon considering its collected form and, of course, is accomplished by distinct authors. Elsewhere, Part II fails to present current researches on the relationship between interoception and interpersonal relationships (social cognition). Part III concludes clinical evidence of interoceptive dysfunctions or illness; however, it pays more attention to neuropathological symptoms and interoception and somewhat neglects possible effective methods of therapeutic interoceptive training. Moreover, in some sense, Part IV deviates from the book's empirically oriented target but falling into philosophical demarcation of generic feelings (even proto-feelings).

Notwithstanding, the book presents many illuminating insights about interoception and cognition. Further, the book will be of interest to those critical of Standard Cognitive Science (see e.g., Shapiro, 2019) and possibly those advocating new strands of cognitive science.

## Author Contributions

JZ and DD wrote the manuscript, with larger contributions by JZ. DD provided edits and suggestions for revision. All authors contributed to the article and approved the submitted version.

## Conflict of Interest

The authors declare that the research was conducted in the absence of any commercial or financial relationships that could be construed as a potential conflict of interest.
